# Correlation of lifestyle behaviors during pregnancy with postpartum depression status of puerpera in the rural areas of South China

**DOI:** 10.3389/fpubh.2023.1304226

**Published:** 2023-12-18

**Authors:** Ye Ding, Genyuan Li, Xi Shi, Mengyi Wang, Yanxia Peng, Huiqin Deng, Ziqi Yang, Qingfen Liang, Zhixu Wang

**Affiliations:** ^1^Department of Maternal, Child and Adolescent Health, School of Public Health, Nanjing Medical University, Nanjing, China; ^2^Jiaxing Center for Disease Control and Prevention, Jiaxing, China; ^3^Department of Nutrition and Food Hygiene, School of Public Health, Institute of Nutrition, Fudan University, Shanghai, China; ^4^Zijin Maternal and Child Health Hospital, Heyuan, China; ^5^Longchuan Maternal and Child Health Hospital, Heyuan, China; ^6^Tianyang Maternal and Child Health Hospital, Baise, China; ^7^Lingshan Maternal and Child Health Hospital, Qinzhou, China

**Keywords:** depression, postpartum, life style, pregnancy, correlation of data

## Abstract

**Background:**

Postpartum depression (PPD) is among the most common postpartum complications. Its prevalence is associated with strong regional variability. Women in rural areas of China have a high risk of PPD. The aim of this study was to investigate the PPD status of women in rural South China and explore the effects of modifiable lifestyle behaviors during pregnancy on their PPD status, thereby providing a scientific basis for the prevention and intervention of PPD in rural China.

**Methods:**

A cohort study was conducted on 261 women from four maternal health institutions situated in rural areas of Guangdong Province and the Guangxi Zhuang Autonomous Region from October 2021 to December 2022. The questionnaires were administered to these women to obtain data about sociodemographic characteristics, health literacy, physical activity during pregnancy, and sleep and dietary status during pregnancy, as well as depression status on the 42^nd^ day after delivery. The lifestyle behaviors during pregnancy and the PPD status of the study population were analyzed. Multiple linear regression models were used to determine the correlation between lifestyle behaviors and PPD status. Path analysis was performed to explore the interaction between various lifestyle behaviors.

**Results:**

A total of 14.6% of women had a PPD status. Women who continued to work during pregnancy had an Edinburgh Postpartum Depression Scale (EPDS) score of 1.386 points higher than that of women who did not (*В* = 1.386, *β* = 0.141, *p* = 0.029). For every 1-point increase in the infant feeding-related knowledge score and pregnancy diet diversity score, the EPDS score decreased by 0.188 and 0.484 points, respectively, and for every 1-point increase in the Pittsburgh sleep quality index score, the EPDS score increased by 0.288 points. Age was related to infant feeding-related knowledge (indirect path coefficient = 0.023). During pregnancy, sedentary time was correlated with sleep quality (indirect path coefficient = 0.031) and employment status (indirect path coefficient = 0.043).

**Conclusion:**

Employment status, infant feeding-related knowledge, sleep quality, and diet diversity during pregnancy directly influenced the PPD status, while age and sedentary time during pregnancy indirectly influenced the PPD status. Promoting healthy lifestyle behaviors, including reducing sedentary time, improving sleep quality, and increasing dietary diversity, may be effective in reducing PPD occurrence.

## 1 Introduction

Postpartum depression (PPD) is a major psychiatric concern during the puerperium phase and is among the most frequent complications encountered during this phase (1). In 2021, the global PPD prevalence was approximated at 17.2% (2). A comprehensive meta-analysis of 95 studies within the Chinese demographic indicated a PPD prevalence rate of 16.3% (3). PPD has profound, multifaceted implications for global public health. It exerts substantial psychological strain on puerperal women, thereby heightening their susceptibility to additional perinatal disease states (4, 5). Concomitantly, PPD detrimentally affects the maternal–infant bond and influences the child’s physiological, cognitive, emotional, personality, and behavioral adaptation (6–8). Additionally, PPD affects spouses’ emotions and family harmony (9).

Major regional disparities exist in PPD occurrence across diverse spatial demographics (10). With China’s urbanization progressing expediently and living standards augmenting markedly, rural inhabitants are grappling with persistent challenges ranging from depressed income, limited educational attainments, antiquated mindsets, inadequate transportation frameworks, and a paucity of essential medical resources, including deficient mental health services (11, 12). These collective factors can potentially hinder the effective detection and intervention of PPD within the female rural populace, thereby inciting grave consequences. Empirical research corroborates a higher degree of PPD prevalence among rural puerpera, which is a stark contrast to their urban counterparts (13). The elevated incidence of maternal PPD within rural territories of China necessitates urgent attention.

Currently, the most empirical discourse related to PPD is focused on validating causative factors (14), while identifying protective elements represents a pivotal approach in mitigating PPD risk. Studies have acknowledged the associations between PPD and antecedents such as mental health history (15), biological determinants (16, 17), socioeconomic variables (18, 19), COVID-19 pandemic (20), obstetric chronicles (21), and lifestyle behaviors. Notably, the impact of modifiable lifestyle behaviors, such as health literacy, physical activity, and sleep and dietary quality, maternal psychological health has been demanding increasing attention. For example, a survey conducted by Zhu Y et al. on postpartum women on the 42^nd^ day after delivery found that maternal and infant health literacy was a protective factor for PPD (22). Through a multiethnic group in Oslo, Shakel N et al. found that women who met the recommendations for physical activity during pregnancy had a lower risk of PPD status compared to women who were inactive during pregnancy (23). Similarly, through a cohort study, Gao M et al. found that Poor sleep quality in the second trimester among Chinese pregnant women was associated with stress and depression symptoms (24). Yang C et al. found that poor dietary quality during lactation was associated with PPD in Chinese lactating women (25). However, these few available studies have adopted a unidimensional perspective, examining the influence of single variables on PPD. In fact, these factors are interdependent and may interact to impact PPD manifestation. Although some studies involved multiple factors, they still did not consider the mutual influence between these factors. For example, physical activity and sleep patterns have been construed as confounding variables in a study investigating the correlation of dietary patterns with PPD (26). Therefore, a perspicacious exploration of the mutual influences of these lifestyle behaviors is warranted.

Most existing studies on PPD determinants are cross-sectional studies (27); therefore, their ability to establish conclusive causality remains limited. This study examined the demographic data, health literacy, physical activity, and sleep and dietary quality, along with other lifestyle behavior metrics of women residing in rural parts of southern China, commencing from pregnancy. Additionally, we assessed the PPD status at 42 days postpartum, investigated the correlation between these potential influencing elements and PPD manifestation, and analyzed inter-relationships among these factors, with a goal to uncover risk variables for PPD. We intend to identify high-risk demographics within rural China during pregnancy for early mental health counseling and intervention, thus ensuring optimal maternal and neonatal mental health.

## 2 Methods

### 2.1 Study design and participants

From October 2021 to December 2022, the recruitment of participants was carried out using a multi-stage sampling method. Firstly, based on the geographical location and convenience of implementation, Guangdong Province and Guangxi Zhuang Autonomous Region were selected as representatives in southern China. Then, each province (autonomous region) was divided into urban and rural areas. Finally, two maternal and child health institutions were randomly selected from rural areas of each province (autonomous region). Pregnant women aged 18–49 who had resided locally for more than 12 months were recruited at these hospitals. Women with speech communication difficulties or mental disorders were excluded. The study was approved by the Medical Ethics Committee of Tongji Medical College, Huazhong University of Science and Technology (No. 2021-S092), and written informed consent was obtained from all participants.

**Figure 1:**
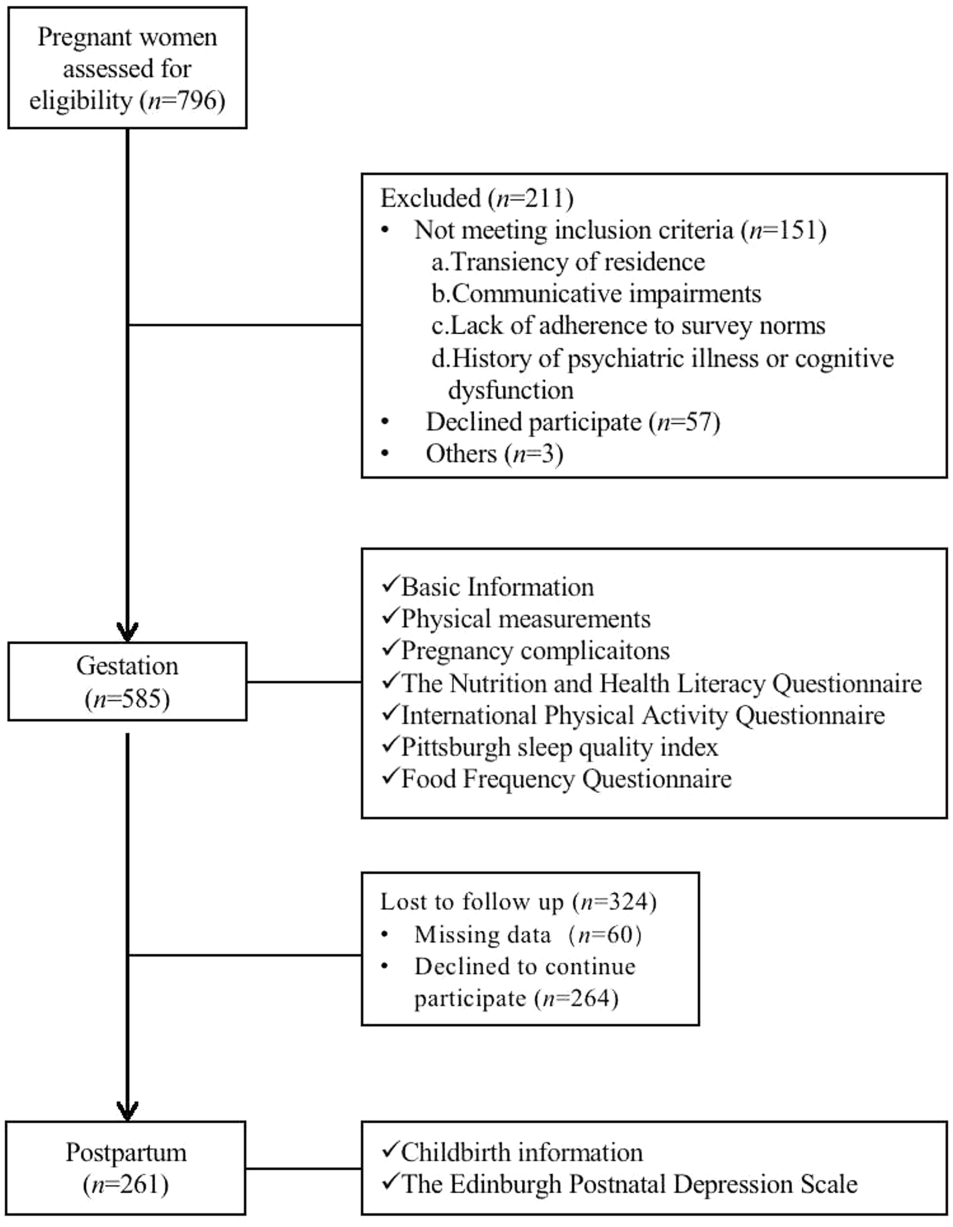
The flow chart of the study participants.

The flow chart of the cohort study was shown in Figure 1. During pregnancy, the sociodemographic characteristics, physical activities, and sleep and dietary quality of the participants were collected through face-to-face interviews, while their PPD status on the 42^nd^ day after delivery were recorded using self-reporting questionnaires.

The sample size of our study was determined using the formula: $n=\frac{Z_{\alpha }^{2}\times P\left(1-P\right)}{\delta^{2}}$. *Ζ* is the value associated with the desired confidence level, and the confidence level was set at 95% (*Z*_α_ = 1.96) in our study. δ is the maximum permissible error allowed by us, which is 0.05. *P* is the prevalence of PPD among Chinese women. In our study, this value was the result of a meta-analysis of 24 studies conducted in China, with a value of 15.0% (10). It was calculated that at least 196 participants were required for this study. This study enrolled a cohort of 261 women, which met our expectations.

### 2.2 Data collection

The Nutrition and Health Literacy Questionnaire is developed by our research group specifically for evaluating the health literacy of Chinese pregnant women, and was determined by authoritative experts based on the importance of the items. It has an internal consistency Cronbach’s α coefficient of 0.719 and a Kaiser–Meyer–Olkin Measure (KMO) value of 0.771. Bartlett’s test of sphericity was significant at *p* < 0.001, which thus verified the structural validity of this questionnaire. The questionnaire is divided into three domains: foundational knowledge and concepts, lifestyle and behavior, and fundamental skills, with domain containing varying topics related to nutritional knowledge, nutrition and diseases, lifestyle, infant feeding-related knowledge, weight management, disease management, and information recognition and decision. It encompasses a total of 24 items, with aggregate scores of 0–96 points.

The physical activity status of pregnant women during the preceding week was assessed using the Chinese version of the International Physical Activity Questionnaire-Short Form (IPAQ-S) (28). The IPAQ-S has been widely used in the Chinese population and has shown favorable reliability and validity. The agreement between the IPAQ-S and the 24-h physical activity records was more than 70% (28). This questionnaire evaluated the frequency and daily durations of three categories of physical activities, namely ambulation, moderate intensity, and high intensity, conducted over nearly a week, and the total time dedicated to sedentary behaviors. The metabolic equivalent (MET) values were designated, followed by the computation of physical activity intensities for all three aforementioned categories. The physical activity intensity of a specific level (MET-min/w) was calculated as follows: The MET value assigned to that intensity of physical activity × weekly frequency (d/w) × daily duration (min/d). The aggregate physical activity intensity for a participant was the summation of the intensity levels from all three modes. Based on the parameters suggested by the IPAQ Working Group, overall physical activity intensity levels in pregnant women were categorized as high, moderate, and low (29).

The Chinese version of the Pittsburgh sleep quality index (PSQI) was used to evaluate the sleep quality of pregnant women in the preceding month (30). This Chinese version of the PSQI tool has been validated for use in the Chinese population, with an internal consistency Cronbach’s α coefficient of 0.845 (30). The questionnaire comprises 18 items and is structured into seven subcategories: subjective sleep quality, sleep latency, sleep duration, sleep efficiency, sleep disturbances, use of sleeping medication, and daytime dysfunction. Each subcategory is rated on a scale from 0 to 3, with the cumulated score of all seven subcategories representing the overall PSQI score. The overall score extends from 0 to 21, and a higher score denotes lower sleep quality. A total score of ≤5 signifies good sleep quality, a total score of 6–10 implies general sleep quality and a score of ≥11 denotes poor sleep quality (31).

The semi-quantitative Food Frequency Questionnaire (FFQ), tailored specifically for Chinese pregnant women, was used to determine the food intake frequency among the pregnant women over the preceding month. This FFQ is recognized for its validity and reliability. For foods, the intraclass correlation coefficients of two administrations of FFQ ranged from 0.23 (nuts) to 0.49 (fruits), and the energy-adjusted and de-attenuated correlation coefficients between FFQ and three 24-h dietary recalls ranged from 0.35 (beans) to 0.56 (fruits) (32). The Dietary Diversity Score (DDS), computed from the obtained dietary information, is used to assess dietary quality during gestation (33). As per the Dietary Guidelines for Chinese Residents 2022 (34), food intake is segregated into 11 categories: cereals and their products, potatoes, vegetables, fruits, poultry, livestock, aquatic products, eggs, milk/dairy products, soybeans/products, and nuts. Each unique category of food ingested in 24 h was assigned one point, with no additional points being assigned for repetitive consumption. Derived from a cumulative sum, the DDS score is a comprehensive measure of overall dietary quality. In this study, items containing high levels of oil, salt, and sugar, such as fried foods, pickled products, and sugary beverages, were not included in the DDS scoring parameters. The total DDS score varies between 0 and 11 points.

The Chinese Version of the Edinburgh Postnatal Depression Scale (EPDS), initially developed by Cox et al. in 1978 and subsequently translated and revised by Wang Yuqiong et al. in 2009, was employed to evaluate the PPD status (35, 36). The scale has an internal consistency Cronbach’s α coefficient of 0.790 and a Guttman’s split-half coefficient of 0.760, indicating favorable reliability and validity (36). The scale comprises 10 items, each of them grading across four levels and assigned scores ranging from 0 to 3. The cumulative score of the 10 items yields the EPDS score of 0–30. An EPDS score of ≥13 is indicative of PPD status, with higher scores denoting more severe depression.

### 2.3 Statistical analysis

The normality of continuous variables was tested before analysis. Normally distributed continuous variables were expressed as the mean ± standard deviation (SD) and were compared using the independent sample t-test. Non-normally distributed continuous variables were expressed as median (*P*_25_, *P*_75_) and were compared using the Mann–Whitney *U* rank sum test. Categorical variables were expressed as frequency (*n*) and percentage (%) and analyzed using the Chi-square test (*χ*^2^). The multiple linear regression was employed to investigate the correlation between lifestyle behaviors factors (including health literacy, physical activity, and sleep and dietary status) during pregnancy and postpartum PPD status. As a valuable technique for dissecting the direct correlation between lifestyle behaviors and PPD status, the path analysis was performed to assess the direct and indirect significance of independent variables on the dependent variable. Statistical analysis of all data was performed using SPSS 26.0, Results were considered to be statistically significant for *p* < 0.05.

## 3 Results

### 3.1 Sociodemographic characteristics

14.6% of 261 women had a PPD status. The average age of the participants was 30.7 ± 5.5 years. The average ages in the PPD and non-PPD groups were of 29.3 ± 4.5 and 31.0 ± 5.7 years, respectively. The between-group comparison revealed a statistically significant difference (*p* = 0.044). Regarding employment status during pregnancy, the proportion of women who did not engage in work was significantly lower in the PPD group (42.1%) than in the non-PPD group (71.7%; *p* < 0.001). No significant differences were observed in other sociodemographic characteristics between the two groups (Table 1).

**Table 1:** Sociodemographic characteristics of the women in two study groups.

Characteristics	Total (*n* = 261)	PPD (*n* = 38)	Non-PPD (*n* = 223)	*t*/*χ*^2^	*P*
Age (years), mean ± SD	30.7 ± 5.5	29.3 ± 4.5	31.0 ± 5.7	−2.059	**0.044**
BMI (kg/m^2^), mean ± SD	24.4 ± 3.6	23.8 ± 3.5	24.5 ± 3.7	−1.049	0.295
Ethnicity, *n* (%)				4.119	0.053
Han	132 (50.6)	25 (65.8)	107 (48.0)		
Minority	129 (49.4)	13 (34.2)	116 (52.0)		
Educational level, *n* (%)				4.628	0.199
Junior high school and below	100 (38.3)	9 (23.7)	91 (40.8)		
Senior high school/vocational high school	58 (22.2)	10 (26.3)	48 (21.5)		
Junior college/vocational university	56 (21.5)	9 (23.7)	47 (21.1)		
Bachelor’s degree and above	47 (18.0)	10 (26.3)	37 (16.6)		
*Per capita* monthly income (RMB), *n* (%)				6.119	0.051
<3,000	114 (43.7)	12 (31.6)	102 (45.8)		
3,000–5,000	68 (26.0)	16 (42.1)	52 (23.3)		
>5,000	79 (30.3)	10 (26.3)	69 (30.9)		
Employment, *n* (%)				12.992	**< 0.001**
Unemployed	176 (67.4)	16 (42.1)	160 (71.7)		
Employed	85 (32.6)	22 (57.9)	63 (28.3)		
Pregnancy complications, *n* (%)				0.092	0.856
No	166 (63.6)	25 (65.8)	141 (63.2)		
Yes	95 (36.4)	13 (34.2)	82 (36.8)		
Mode of conception, *n* (%)				0.926	0.336
SP	250 (95.8)	38 (100.0)	212 (95.1)		
ARTP	11 (4.2)	0 (0.0)	11 (4.9)		
Parity, *n* (%)				0.794	0.420
Primiparous	198 (75.9)	31 (81.6)	167 (74.9)		
Multiparous	63 (24.1)	7 (18.4)	56 (25.1)		
Gestational age at birth, *n* (%)				0.616	0.433
Preterm	11 (4.2)	3 (7.9)	8 (3.6)		
Term	250 (95.8)	35 (92.1)	215 (96.4)		
Mode of delivery, *n* (%)				2.177	0.186
Spontaneous labor	178 (68.2)	22 (57.9)	156 (70.0)		
Cesarean section	83 (31.8)	16 (42.1)	67 (30.0)		

PPD, postpartum depression; SP, spontaneous pregnancy; ARTP, assisted reproductive technology pregnancy.

### 3.2 Correlation of lifestyle behaviors with PPD status during pregnancy

The medians of the total health literacy score of the PPD and non-PPD groups were 35.0 and 40.0, respectively, and the difference was statistically nonsignificant (*p* = 0.119). Additionally, the two groups differed significantly in the infant feeding-related knowledge score (*p* = 0.035), but no statistically significant differences were observed in other domains and items (Table 2).

**Table 2:** Correlation of health literacy during pregnancy with the PPD status.

	Total (*n* = 261)	PPD (*n* = 38)	Non-PPD (*n* = 223)	*Z*	*P*
Total health literacy score	40.0 (28.0, 48.0)	35.0 (27.0, 48.0)	40.0 (28.0, 50.0)	−1.559	0.119
Foundational knowledge and concepts	8.0 (4.0, 12.0)	8.0 (4.0, 12.0)	8.0 (4.0, 12.0)	−1.125	0.262
Nutritional knowledge	4.0 (0.0, 8.0)	4.0 (0.0, 5.0)	4.0 (0.0, 8.0)	−1.668	0.096
Nutrition and diseases	4.0 (0.0, 8.0)	4.0 (0.0, 5.0)	4.0 (0.0, 8.0)	−0.116	0.922
Lifestyle and behavior	12.0 (12.0, 16.0)	12.0 (8.0, 16.0)	12.0 (12.0, 16.0)	−1.656	0.098
Lifestyle	8.0 (4.0, 8.0)	8.0 (4.0, 8.0)	8.0 (4.0, 8.0)	−0.353	0.729
Infant feeding-related knowledge	8.0 (4.0, 8.0)	4.0 (4.0, 8.0)	8.0 (4.0, 8.0)	−2.112	**0.035**
Fundamental skills	16.0 (12.0, 24.0)	16.0 (12.0, 22.0)	18.0 (12.0, 24.0)	−1.224	0.222
Weight management	4.0 (4.0, 8.0)	4.0 (4.0, 8.0)	4.0 (4.0, 8.0)	−0.075	0.942
Disease management	0.0 (0.0, 4.0)	1.0 (0.0, 4.0)	0.0 (0.0, 4.0)	−0.415	0.678
Information recognition and decision	10.0 (6.0, 14.0)	8.0 (4.0, 13.0)	10.0 (6.0, 14.0)	−1.546	0.123

PPD, postpartum depression. Data are presented as the median values (P_25_, P_75_). The bold values in table means that *p* < 0.05.

The median levels of total physical activity in the PPD and non-PPD groups were 1386.0 and 1485.0 MET-min/w, respectively, and the difference was statistically nonsignificant. Notably, a majority of pregnant women in both groups displayed moderate levels of physical activity. In the PPD group, a higher percentage of pregnant women were classified as having low (23.7%) rather than high (21.0%) levels of physical activity. Conversely, in the non-PPD group, a greater proportion of pregnant women were classified as having high (22.9%) rather than low (16.1%) levels of physical activity. Interestingly, no statistically significant difference was observed in the physical activity classification between the two groups. Further analysis revealed a statistically significant difference (*p* = 0.011) in sedentary time between the PPD and non-PPD groups. The PPD group exhibited a significantly higher median sedentary time than the non-PPD group (280.0 vs. 230.0 min/d; Table 3).

**Table 3:** Correlation of the physical activities during pregnancy with PPD status.

	Total (*n* = 261)	PPD (*n* = 38)	Non-PPD (*n* = 223)	*Z*/*x*^2^	*P*
Physical activity score (MET-min/w)	1386.0 (693.0, 2780.0)	1386.0 (594.0, 2774.0)	1485.0 (693.0, 2919.0)	−0.686	0.495
Physical activity levels, *n* (%)				1.295	0.535
High	59 (22.6)	8 (21.0)	51 (22.9)		
Moderate	157 (60.2)	21 (55.3)	136 (61.0)		
Low	45 (17.2)	9 (23.7)	36 (16.1)		
Sedentary time (min/d)	240.0 (180.0, 300.0)	280.0 (180.0, 332.5)	230.0 (150.0, 300.0)	−2.538	**0.011**

PPD, postpartum depression. Data are presented as the median values (P_25_, P_75_). The bold values in table means that *p* < 0.05.

The median of the PSQI score was significantly higher for the PPD group (7.0) than for the non-PPD group (5.0; *p* = 0.009). In the PPD group, 60.5% of the participants had general sleep quality and 7.9% had poor sleep quality during pregnancy. In the non-PPD group, 54.7% of the participants had good sleep quality and 5.4% had poor sleep quality during pregnancy. The two groups differed significantly in the grading of sleep quality during pregnancy (*p* = 0.023). Moreover, the two groups differed significantly in the dimensions of subjective sleep quality (*p* = 0.014) and sleep latency (*p* = 0.045), whereas the two groups exhibited no statistically significant differences in the other sleep-related dimensions (*p* > 0.05; Table 4).

**Table 4:** Correlation of sleep quality during pregnancy with PPD status.

	Total (*n* = 261)	PPD (*n* = 38)	Non-PPD (*n* = 223)	*Z*/*x*^2^	*P*
PSQI score	5.0 (4.0, 8.0)	7.0 (5.0, 9.0)	5.0 (3.0, 8.0)	−2.601	**0.009**
Sleep quality, *n* (%)				7.252	**0.023**
Good	134 (51.3)	12 (31.6)	122 (54.7)		
General	112 (42.9)	23 (60.5)	89 (39.9)		
Poor	15 (5.8)	3 (7.9)	12 (5.4)		
Sleep-related dimensions					
Subjective sleep quality	1.0 (1.0, 1.0)	1.0 (1.0, 2.0)	1.0 (1.0, 1.0)	−2.445	**0.014**
Sleep latency	1.0 (1.0, 2.0)	2.0 (1.0, 2.0)	1.0 (1.0, 2.0)	−2.003	**0.045**
Sleep duration	0.0 (0.0, 1.0)	1.0 (0.0, 2.0)	0.0 (0.0, 1.0)	−1.089	0.277
Sleep efficiency	0.0 (0.0, 1.0)	1.0 (0.0, 1.3)	0.0 (0.0, 1.0)	−1.629	0.101
Sleep disturbances	1.0 (1.0, 1.0)	1.0 (1.0, 1.3)	1.0 (1.0, 1.0)	−0.563	0.559
Use of sleeping medication	0.0 (0.0, 0.0)	0.0 (0.0, 0.0)	0.0 (0.0, 0.0)	0.000	1.000
Daytime dysfunction	1.0 (0.0, 2.0)	1.0 (0.0, 2.0)	1.0 (0.0, 2.0)	−1.256	0.211

PPD, postpartum depression; PSQI, Pittsburgh sleep quality index. Data are presented as the median (P_25_, P_75_). The bold values in table means that *p* < 0.05.

The medians of DDS score during pregnancy of both the PPD and non-PPD groups were 5.0, while the differences were statistically significant (*p* = 0.014). The weekly intake frequency of different food categories of the two groups showed no statistically significant differences (Table 5).

**Table 5:** Correlation of dietary quality during pregnancy with PPD status.

	Total (*n* = 261)	PPD (*n* = 38)	Non-PPD (*n* = 223)	*Z*	*P*
DDS scores	5.0 (4.0, 6.0)	5.0 (4.0, 5.0)	5.0 (4.0, 6.0)	−2.455	**0.014**
Weekly intake frequency					
Cereals and their products	22.0 (21.0, 24.6)	22.9 (20.6, 26.0)	22.0 (21.0, 24.5)	−0.403	0.688
Potatoes	1.0 (0.0, 2.0)	1.0 (0.0, 2.0)	1.0 (0.0, 2.0)	−0.931	0.354
Vegetables	21.0 (16.8, 24.0)	20.1 (16.9, 24.3)	21.0 (16.6, 24.0)	−0.038	0.970
Fruits	10.0 (7.0, 14.0)	10.0 (7.0, 14.0)	9.4 (7.0, 14.0)	−0.176	0.861
Poultry	14.0 (14.0, 16.5)	14.0 (14.0, 21.0)	14.0 (14.0, 16.0)	−0.561	0.576
Livestock	2.0 (1.0, 3.0)	2.0 (1.0, 3.0)	2.0 (1.0, 3.0)	−0.146	0.885
Aquatic products	2.5 (1.3, 4.0)	2.3 (1.4, 3.8)	2.8 (1.3, 4.0)	−0.353	0.725
Eggs	4.0 (2.5, 7.0)	3.0 (2.0, 7.0)	4.5 (2.3, 7.0)	−1.422	0.156
Milk/dairy products	7.0 (2.0, 7.0)	5.6 (1.2, 7.0)	7.0 (2.0, 7.0)	−1.092	0.276
Soybean/products	2.5 (1.0, 4.0)	2.1 (1.8, 4.0)	2.8 (1.0, 4.0)	−0.088	0.931
Nuts	0.8 (0.0, 6.0)	0.4 (0.0, 5.0)	1.0 (0.0, 7.0)	−0.589	0.558
High-oil, high-salt, and high-sugar food	1.0 (0.0, 3.0)	1.3 (0.3, 4.2)	1.0 (0.0, 2.7)	−1.499	0.134

PPD, postpartum depression; DDS, dietary diversity score. Data are presented as the median value (P_25_, P_75_). The bold values in table means that *p* < 0.05.

### 3.3 Multivariate analysis

To explore the impact of various factors on PPD status, multiple linear regression models were employed. Model 1 incorporated the infant feeding-related knowledge score, sedentary time, PSQI score, and DDS score as independent variables, whereas the EPDS total score was considered as the dependent variable. Building upon Model 1, Model 2 introduced two additional sociodemographic characteristics variables, namely age and employment status during pregnancy, to further investigate their influences.

The results revealed that employment status during pregnancy, infant feeding-related knowledge, PSQI, and DDS scores all affected PPD status. The PPD state can be explained by 8.2% (*R*^2^ = 0.082) of these four variables (Table 6). In Model 2, pregnant women who continued to work during pregnancy had an EPDS score of 1.386 points higher than that of pregnant women who did not work during pregnancy. The EPDS scores decreased by 0.188 and 0.484 points for every 1-point increase in the infant feeding-related knowledge and DDS scores, respectively. For every 1-point increase in the PSQI score during pregnancy, the EPDS score increased by 0.288 points. The sedentary time during pregnancy did not affect the EPDS score (*В* = 0.002, *β* = −0.065, *p* = 0.314). The variance inflation factor in Model 2 was all less than 5, and no collinearity was observed between the variables.

**Table 6:** Multiple linear regression analysis of factors influencing PPD status.

		*В*	SE	*β*	*t*	*P*	VIF	*R* ^2^
Model 1 (*n* = 261)	(Constant)	8.885	1.353		6.567	< 0.001		0.063
	Infant feeding-related knowledge	−0.177	0.080	−0.134	−2.202	**0.029**	1.012	
	Sedentary time	−0.001	0.002	−0.022	−0.357	0.721	1.036	
	PSQI score	0.289	0.099	0.180	2.919	**0.004**	1.044	
	DDS score	−0.460	0.209	−0.134	−2.199	**0.029**	1.015	
Model 2 (*n* = 261)	(Constant)	10.302	1.967		5.236	< 0.001		0.082
	Infant feeding-related knowledge	−0.188	0.081	−0.143	−2.315	**0.021**	1.052	
	Sedentary time	−0.002	0.002	−0.065	−1.009	0.314	1.142	
	PSQI score	0.288	0.098	0.180	2.925	**0.004**	1.047	
	DDS score	−0.484	0.208	−0.141	−2.325	**0.021**	1.020	
	Age	−0.041	0.051	−0.049	−0.804	0.422	1.031	
	Employed	1.386	0.630	0.141	2.201	**0.029**	1.128	

PPD, postpartum depression; PSQI, Pittsburgh sleep quality index; DDS, dietary diversity score; VIF, variance inflation factor. The bold values in table means that *p* < 0.05.

### 3.4 Path analysis

The path coefficient revealed the effect of lifestyle behaviors on PPD status and was calculated according to Model 2. The direct path coefficient exhibited the direct impact of lifestyle behaviors on the PPD status. Among the four independent variables, sleep quality had the most significant influence on the PPD status (0.180), followed closely by the infant feeding-related knowledge score (−0.143). The indirect path coefficient exhibited the influence of independent variables on the dependent variable through other independent variables. A substantial indirect effect of sleep quality was observed on the PPD status through diet quality during pregnancy (0.014) (Table 7).

**Table 7:** Path coefficients of lifestyle behaviors during pregnancy on PPD status.

	Simple correlation coefficient with PPD	Direct path coefficient	Indirect path coefficient					
			Infant feeding-related knowledge	PSQI score	DDS score	Sedentary time	Age	Employed
Infant feeding-related knowledge	−0.133	−0.143	/	0.010	0.010	−0.004	0.008	0.015
PSQI score	0.156	0.180	0.008	/	0.014	0.011	0.003	0.012
DDS score	−0.126	−0.141	0.010	0.018	/	0.002	0.002	0.012
Sedentary time	0.012	−0.065	−0.008	0.031	0.005	/	0.001	0.043
Age	−0.061	−0.049	0.023	0.011	0.006	0.001	/	0.007
Employed	0.107	0.141	0.015	0.016	0.012	0.020	0.002	/

PPD, postpartum depression; PSQI, Pittsburgh sleep quality index; DDS, dietary diversity score.

**Figure 2:**
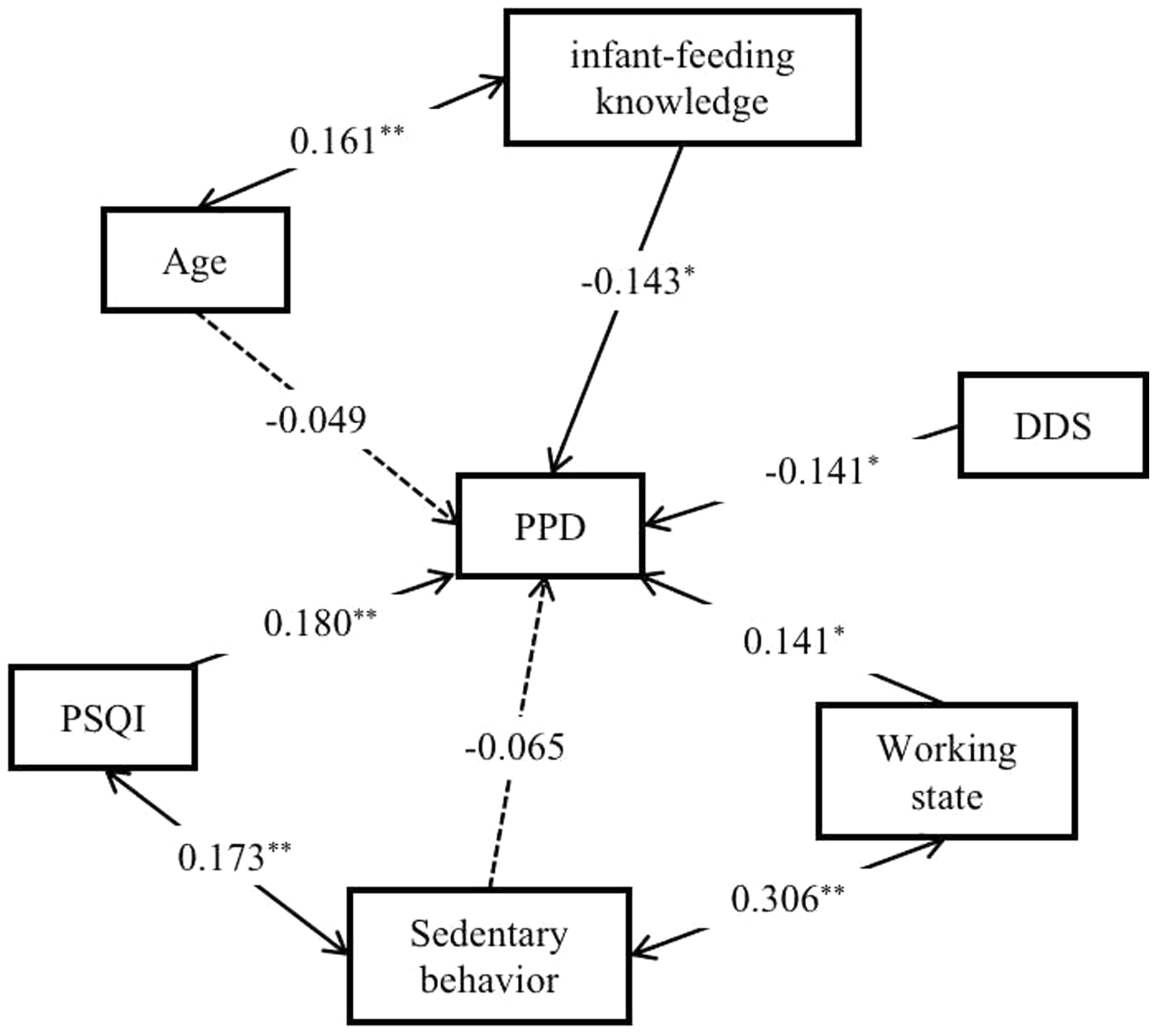
Path analysis of the correlation between lifestyle behaviors during pregnancy and PPD status. ^**^p < 0.01, ^*^p < 0.05. The single arrow line is the direct path line, double arrow line is the correlation line. The solid line represents a significant path coefficient, while the dashed line represents an insignificant path coefficient.

The path analysis diagram (Figure 2) illustrates the relationships among variables. The single-arrow lines depict direct paths, indicating that infant feeding-related knowledge, diet quality, sleep quality, and employment status during pregnancy directly influence PPD development. The double-arrow lines represent correlations. For instance, age is associated with infant feeding-related knowledge during pregnancy, which in turn indirectly affects the PPD status (indirect path coefficient = 0.023). During pregnancy, sedentary time is related to sleep quality and employment status, which means it ultimately has an impact on the PPD status through sleep quality (indirect path coefficient = 0.031) and employment status during pregnancy (indirect path coefficient = 0.043).

## 4 Discussion

This study assessed PPD prevalence in the rural areas of South China. We innovatively shifted the focal point to the prenatal period to examine the impact of four modifiable lifestyle behaviors on the PPD status and to evaluate potential interactions among these lifestyle behaviors. The prevalence of PPD was 14.6% among 261 women. Continuing to work during pregnancy, poor infant feeding-related knowledge, compromised sleep quality, and suboptimal dietary quality are risk factors for PPD. Furthermore, infant feeding-related knowledge during pregnancy mediated the influence of age on PPD. While the extent of sedentary time during pregnancy had a consequential influence on PPD, and this influence was mediated by sleep quality and employment status.

An influx of research on PPD has recently been witnessed within China, however, with considerably divergent findings caused by variations in study design, measurement instruments, sample size, geographical location, and survey timing. We found that 14.6% of puerpera in rural South China reflected a PPD status, a rate lower than that cited in rural Chongqing from 2014 to 2016 (24.10%) (37), yet higher than the rates from selective urban locales in China, for instance, the detection rates in Shenzhen in 2016 (11.5%) (38) and Beijing in 2020 (5.2%) (39). This trend underscores that the Chinese government must allocate increased resources toward mental health considerations among the rural puerpera. This encompassing support should comprise the provision of robust medical resources and expanded opportunities for health education, thereby driving sustained improvements in their mental well-being.

Health literacy, defined as an individual’s capacity to access, comprehend, and use key health information and services, is a pivotal player in health maintenance and promotion (40). Despite its significance, research focused on the intersection of health literacy and PPD is currently scarce. A Vietnamese study reported that pregnant women with higher scores of health literacy had lower likelihood of depression (OR = 0.96; 95%CI: 0.91–0.99) (41). Similarly, a Chinese study identified that the maternal health literacy was a protective factor for PPD (OR = 1.95; 95%CI: 1.26–3.01) (22). Our study, however, did not determine the correlation between the aggregate health literacy score during pregnancy and the PPD status. Instead, a deficient infant feeding-related knowledge acted as a risk factor for PPD, and this relationship was statistically significant even after adjustments were made for confounders. The infant feeding-related knowledge item primarily evaluates the knowledge of pregnant women about feeding their newborns. Infant health can significantly influence PPD, and thus, infant feeding-related knowledge can reflect a mother’s attentiveness toward infant health. According to our findings, a comprehensive understanding of infant feeding-related knowledge could alleviate concerns about infant health concerns, thereby reducing the risk of depression. As such, conducting informational classes and disseminating lectures on maternal nutrition or infant feeding in rural China could facilitate the amplification of health literacy among pregnant women, potentially diminishing PPD susceptibility.

The influence of physical activity on PPD is underscored by a spectrum of conflicting evidence. According to Shakeel et al., pregnant women undertaking at least 150 cumulative minutes of moderate to vigorous physical activity per week, with each session lasting more than 10 min, had a significantly reduced risk of PPD (OR = 0.2, 95% CI: 0.06–0.63) (23). By contrast, Susukida et al. suggested that physical activities during pregnancy, compared with the absence of physical activities, had heightened the risk of PPD development by 42.0% (AOR: 1.42, 95% CI: 1.24–1.61) (42). Thiel’s cohort study yielded neutral findings, indicating no apparent link between physical activity during pregnancy and PPD (43). Our study also failed to authenticate an association between total physical activity during pregnancy and PPD status. This variability could be because low-to-moderate physical activity levels were predominant during pregnancy, observed in 77.4% of our study population. Complementarily, although sedentary time does not directly influence the PPD status, we evaluated sedentary time, which was associated with the significant determinants of PPD, that is, sleep quality and employment status during pregnancy. Corroborating our findings, a Brazilian adult cohort revealed that those with daily sedentary time exceeding 10 h were more susceptible to depressive symptoms than those who sat for less than 10 h (44).

Postpartum sleep quality robustly impacts PPD incidence. Previous studies hypothesized that poor sleep quality was significantly associated with greater symptoms of PPD (45). Nonetheless, most of these studies have employed cross-sectional designs, thereby introducing challenges in ascertaining the directional causality of this relationship. Some researchers have preponed the observational time point to gestation to investigate the relationship between sleep quality and PPD. In a cohort aged under 30 years, some researchers observed an affirmative correlation between the PSQI during gestation and the EPDS score (*β* = 0.51, 95% CI: 0.38–0.64) (24). Similarly, Gueron-Sela et al. highlighted that sleep duration throughout pregnancy correlates with PPD severity; shorter sleep spans during pregnancy were associated with a more pronounced risk of severe PPD (46). The present study identified a similar trend; higher PSQI scores that indicate deteriorating sleep quality during pregnancy corresponded with elevated EPDS scores (*В* = 0.288, *β* = 0.180, *p* = 0.004).

Mounting evidence has highlighted the significance of diet as a pivotal and modifiable lifestyle behavior influencing PPD. Numerous studies have underscored that postpartum diet quality is inversely correlated with PPD symptoms (25). Nonetheless, studies investigating the impact of diet quality during pregnancy on PPD are limited. The DDS is an assessment criterion for dietary quality and is also used for measuring eating experiences (47). This study discovered that the overall dietary quality of pregnant women residing in the rural regions of southern China is poor, with a lower DDS score identified as a risk factor for PPD. After adjustments were made for the common linear effects of the DDS score and other influential factors, each 1-point elevation in the DDS score during pregnancy corresponded to a 0.484-point decrease in the EPDS. Consistent with our findings, Poorrezaeian et al. delineated that each unit increase in the DDS score had a 39% protective effect against major depression in adult women (48). However, in a study including Spanish adults, dietary diversity was significantly and inversely associated with depression prevalence, yet no correlation was observed regarding changes in depression over 2 years (49). This suggested that dietary diversity may not necessarily mitigate the depression risk. Studies investigating the link between dietary diversity and PPD are currently scarce. Extensive longitudinal studies with prolonged follow-up durations are warranted to comprehensively evaluate the impact of dietary diversity on PPD.

Various lifestyle behaviors often engage in interconnected dynamics. For instance, pregnant women following a nutritious dietary regimen tend to demonstrate greater physical activity, while those exhibiting superior health literacy often incline toward healthier meal choices (50). Furthermore, a substantiated correlation has been established between diet quality and sleep quality. The intake of healthy foods was related to better sleep quality, while the more processed and free-sugar rich foods were consumed, the poorer the sleep features (51). Van Lee et al. examined the cumulative effect of multiple risk factors on perinatal depression and found that among six risk factors, namely poor dietary quality, impaired sleep quality, insufficient physical activity, Vitamin D deficiency, smoking during pregnancy, and lack of social support, pregnant women with four or more of the risk factors exhibited a six-fold higher risk of antenatal depression than those with one or fewer of the risk factors (52). Our study similarly identified correlations between various lifestyle behaviors. Maternal age exhibited a meaningful correlation with infant feeding-related knowledge during pregnancy. Older pregnant women possess extensive experiential wisdom and a comprehensive understanding of gestation and neonatal care. During pregnancy, sedentary time was associated with sleep quality and employment status. Women who continued to work during pregnancy, affected by work-related factors, tended toward more sedentary time than those not working during pregnancy. Furthermore, occupational stress might influence sleep quality during pregnancy.

This study is associated with certain limitations. Initially, the sample for this study were obtained through convenient sampling and the size was small. Postpartum women navigate not only their convalescent journey but also contribute significantly toward childcare. Consequently, the proportion of women traced after the postpartum period is diminished, potentially introducing selection bias. Second, the lifestyle behaviors were assessed a single time during gestation. However, lifestyle behaviors undergo dynamic alterations during pregnancy. Future studies could find involving multiple temporal evaluation points for identifying the ideal epoch for the initiation of intervention strategies beneficial. At last, contrary to objective assessment instruments and clinical gold standards, the approach of using the EPDS, a subjective self-reporting tool, to investigate maternal depression harbors a degree of subjectivity, thereby engendering a propensity toward bias.

## 5 Conclusion

In summary, myriad factors influence PPD onset, and amidst this context, the present study signifies a promising outset in the domain of investigating maternal PPD in the rural territories of China. Deciphering the interrelations between diverse lifestyle behaviors during gestation could make us aware of optimal interventional blends for PPD during pregnancy aimed at minimizing or obliterating the impact of these risk factors on pregnant women in rural China, thereby reducing PPD susceptibility. Moreover, these results have potential value in identifying risk factors for PPD, developing three-level prevention measures and ensuring the physical and mental health of mothers and infants. For example, delivering apt dietary guidance to rural Chinese pregnant women, rendering improvements in their dietary quality, and attenuating the risk of underconsumption of certain indispensable nutrients are imperative. This in turn could further reduce the likelihood of PPD manifestation. Future studies could leverage devices, such as sports watches and sleep recorders, to objectively evaluate and thus delve deeper into the implications of variations in physical activity and sleep status on PPD.

## Data availability statement

The data that support the findings of this study are available on request from the corresponding authors upon reasonable request, and any personal information that might compromise the privacy of the participants in the data has been deleted.

## Ethics statement

The studies involving human participants were reviewed and approved by the Medical Ethics Committee of Tongji Medical College, Huazhong University of Science and Technology (No. 2021-S092). The participants provided their written informed consent to participate in this study. Written informed consent was obtained from the individual(s) for the publication of any potentially identifiable images or data included in this article.

## Author contributions

YD: Conceptualization, Formal analysis, Methodology, Writing – original draft, Writing – review & editing. GL: Data curation, Formal analysis, Investigation, Writing – original draft, Writing – review & editing. XS: Data curation, Investigation, Writing – original draft. MW: Data curation, Investigation, Writing – original draft. YP: Data curation, Investigation, Writing – original draft. HD: Data curation, Investigation, Writing – original draft. ZY: Data curation, Investigation, Writing – original draft. QL: Data curation, Investigation, Writing – original draft. ZW: Conceptualization, Funding acquisition, Methodology, Supervision, Writing – review & editing.
